# An Impedance-Based Mold Sensor with on-Chip Optical Reference

**DOI:** 10.3390/s16101603

**Published:** 2016-09-28

**Authors:** Poornachandra Papireddy Vinayaka, Sander van den Driesche, Roland Blank, Muhammad Waseem Tahir, Mathias Frodl, Walter Lang, Michael J. Vellekoop

**Affiliations:** 1 Institute for Microsensors, Actuators and Systems (IMSAS), University of Bremen, Otto-Hahn-Allee NW1, Bremen 28359, Germany; sdriesche@uni-bremen.de (S.v.d.D.); rblank@imsas.uni-bremen.de (R.B.); wtahir@imsas.uni-bremen.de (M.W.T.); wlang@imsas.uni-bremen.de (W.L.); mvellekoop@imsas.uni-bremen.de (M.J.V.); 2 Microsystems Center Bremen (MCB), Bremen 28359, Germany; 3 microFAB Service GmbH, Bremen 28359, Germany; frodl@microfab.de

**Keywords:** mold, impedance, colorimetric, culture medium, archives, transport containers

## Abstract

A new miniaturized sensor system with an internal optical reference for the detection of mold growth is presented. The sensor chip comprises a reaction chamber provided with a culture medium that promotes the growth of mold species from mold spores. The mold detection is performed by measuring impedance changes with integrated electrodes fabricated inside the reaction chamber. The impedance change in the culture medium is caused by shifts in the pH (i.e., from 5.5 to 8) as the mold grows. In order to determine the absolute pH value without the need for calibration, a methyl red indicator dye has been added to the culture medium. It changes the color of the medium as the pH passes specific values. This colorimetric principle now acts as a reference measurement. It also allows the sensitivity of the impedance sensor to be established in terms of impedance change per pH unit. Major mold species that are involved in the contamination of food, paper and indoor environments, like *Fusarium oxysporum*, *Fusarium incarnatum*, *Eurotium amstelodami*, *Aspergillus penicillioides* and *Aspergillus restrictus*, have been successfully analyzed on-chip.

## 1. Introduction

Mold is a general terminology for a variety of filamentous fungi. Although molds play a vital role in the decomposition of organic matter present in our ecosystem, some of them are proven to pose a severe threat to human and animal health. They also cause damage to perishable goods, books, buildings, or other organic matter. With the trade markets expanding rapidly between different countries, more attention has been drawn towards designing a remote monitoring system to determine the quality of the goods during and after transportation. Yearly, more than one quarter of produced food is wasted because of a drop in quality during the food supply chain [1]. In food chain management, parameters that affect the quality of food like temperature, humidity, air flow, CO_2_ and ethylene are monitored regularly [2]. However, monitoring the mold contamination of food products is often neglected [3]. Food can be infected with mold before being harvested or during the process of transportation, so it is of the utmost importance to monitor the growth of mold during transportation.

**Figure 1:**
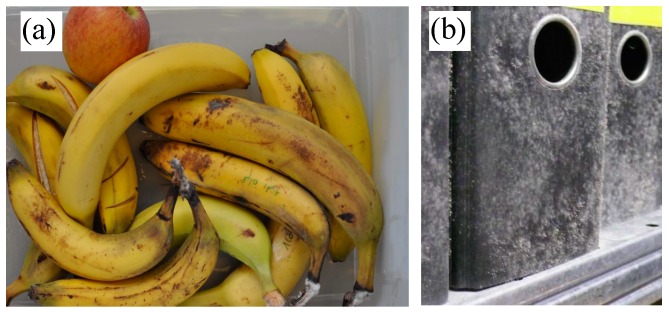
Photos showing mold contamination on bananas (**a**) and on files (**b**). Source: BMA Labor GmbH.

Mold contamination is also a major issue in archives. In most cases, the infestation is due to improper control of climatic conditions. Mold spores enter via air ventilators or by animal interventions and start to germinate, resulting in degradation of papers, bindings, files, wooden roofs, and other organic materials. If mold growth is unnoticed in its initial stage, it could spread through the entire indoor environment. In addition to the product losses generated due to mold infestation, the growth of mold produces mycotoxins and microbial volatile organic compounds (MVOC’s), which are proven to be dangerous to humans and animals [4,5,6]. Considering all of these adverse effects of mold growth, it is necessary to detect the mold contamination in its early stage. Figure 1 shows images of mold-infected bananas (a) and of mold contaminated files in an archive (b).

Current sensor systems for the detection of mold in indoor environments are large, costly, and labor intensive. In this work we propose a low-cost small sensor system that can be easily integrated with an air-sampling unit for on-site monitoring of mold contaminations. 

Different analytical methods are employed for the detection and enumeration of mold [7]. Microscopic mold detection techniques rely on the visual inspection of suspicious samples collected from air or from an infected surface. Using this optical detection principle an automated pollen and spore counting system for outdoor environments was designed and realized [8,9]. However, it does not distinguish the viable microbes from non-viable ones. Having information about the quantity of spores is not sufficient to confirm the risk of mold contamination because only viable spores are responsible for mold growth [10]. 

Cultivation techniques do rely on mold growth. Once a sample is collected and placed on a nutrient medium, viable spores are allowed to grow and form colonies, which are later counted in terms of colony forming units (CFU) for quantification. Since various microorganisms prefer different nutrients essential for their growth it is possible to use a specific culture medium for each type of species. The main disadvantage of traditional culture methods is the time it takes for the species to grow (usually days), forming a visible colony for counting. Image processing techniques allow automated counting of the colonies, which reduces the manual effort for counting mold colonies.

ATP-bioluminescence is an attractive method for the rapid detection of microorganisms [11,12]. Adenosine triphosphate (ATP) is a molecule produced by all living cells and can be quantified by a luciferase based bioluminescence reaction. The milliflex rapid microorganism detection system is a commercial system that uses this bioluminescence principle. Although this method can rapidly determine the amount of biological species present in a sample, to automate this method is complex because the microbes have to be lysed (to release the ATP) and mixed with substrate and enzymes required for the bioluminescence reaction. 

In recent years, molecular methods have received attention for the detection of pathogens present in food or in air, as these methods offer rapid detection times. Immunoassays are based on the measurement of antibody-antigen interactions [13]. Using this principle several biosensors are under development. Though this method is successful, there are complications in selecting the required antibodies with high specificity for the vast variety of the mold species present in our ecosystem. Another molecular method for identification of mold is polymerase chain reaction (PCR), which is based on specific DNA sequences [14]. Although PCR has a good specificity, it is affected by contaminations present in the measurement environment. It needs several laborious sample preparation steps (like purification and extraction of DNA) that require experienced professionals. 

Another well-known method for the detection of pH in a small area is the use of ion-sensitive field effect transistors (ISFET). However, they show some problems with stability [15]. Therefore, along with ISFETs, reference measurements are needed. In addition, manufacturing of the ISFETs requires more complex processes (e.g., diffusion, implantation, etching, etc.) and will be more costly. 

Various spectroscopic techniques such as Fourier transform infrared spectroscopy (FTIR) and mass spectroscopy have been introduced for identification of microorganisms. FTIR is a nondestructive method for identification of microorganisms. Based on the molecular composition, molds absorb infrared light generating a spectrum [16]. Similarly, mass spectroscopy allows in determining a fingerprint of microorganisms by ionization methods [17]. They are used for further comparisons or identification of molds. Bruker Daltonics demonstrated a mold detection instrument (MALDI Biotyper, Bruker Daltonics, EW Leiderdorp, The Netherlands) which is based on mass spectroscopy to identify the molecular fingerprint of several microorganisms. These spectroscopic methods are confined to laboratory use because of their complexity and cost. Also, the sample has to be prepared in a clean environment as these methods are sensitive to contaminants. 

Another possibility for mold detection is by measuring the microbial volatile organic compounds (MVOC’s) released by mold due to their metabolic activities [18,19]. A combination of solid phase micro-extraction and gas chromatography techniques are used to determine the MVOC’s or one can use an electronic nose for analyzing the gases emitted by mold. It was shown that the MVOC pattern emitted by the same mold species could vary depending on the age and climatic conditions in which it is growing, which makes it complex to use this principle for detecting mold.

All these methods are not suitable for designing a miniaturized out-of-lab detection system because they are not stable enough or require several sample preparation steps which cannot be integrated into an autonomous system, or they are expensive. Hence, in this work we propose automating the traditional culturing method for making an autonomous system suitable for out-of-lab mold detection, such as in transport containers or in archives. We use a miniaturized culture medium-based system with integrated reference measurement. The detection principle is a physiochemical method, which is based on the measurement of changes in the electrical impedance of the medium occurring due to the pH change, as the mold grows. The major advantage of a sensor system using culture medium is that it specifically detects the viable molds, which are the main concern for books (in archives) and food (in transportation). In the ultimate system, the spores are brought to the sensor by an integrated air-sampling unit that typically pumps 600 liters per hour. This means that the spores can be detected before they develop into mold on objects such as books or food. 

Culture medium-based impedance sensing methods are also known for the detection of bacterial growth [20,21,22,23]. When bacteria grow on the medium, it will result in an impedance change. Although our sensor is also sensitive for bacteria contamination, we established that for the aimed applications (banana containers and archives), the possible bacteria contamination is very low compared to mold spore contamination. In addition, because the presence of bacteria is also harmful to goods, it is not a problem that the sensor reacts to this. In literature the use of chloramphenicol antibiotic is being described as an effective method to prevent bacteria growth [24]. However, bacteria detection has not been a part of this study.

All the published work applying impedance measurement principles suffer from a lack of reference measurement in the system. Although pH changes can be detected with the electrodes, determination of an absolute pH value from these measurements needs a calibration step. The integration of the colorimetric reference method within the sensor allows the determination of the pH value and in addition the sensor´s sensitivity (impedance change per unit pH) without the need of calibration [25,26]. This approach is applied for the detection and quantification of *Fusarium oxysporum*, *Fusarium incarnatum*, *Eurotium amstelodami*, *Aspergillus penicillioides* and *Aspergillus restrictus*, molds that are very common in archives and food transport. 

## 2. Materials and Methods 

### 2.1. Impedance Sensor and Optical Reference

**Figure 2:**
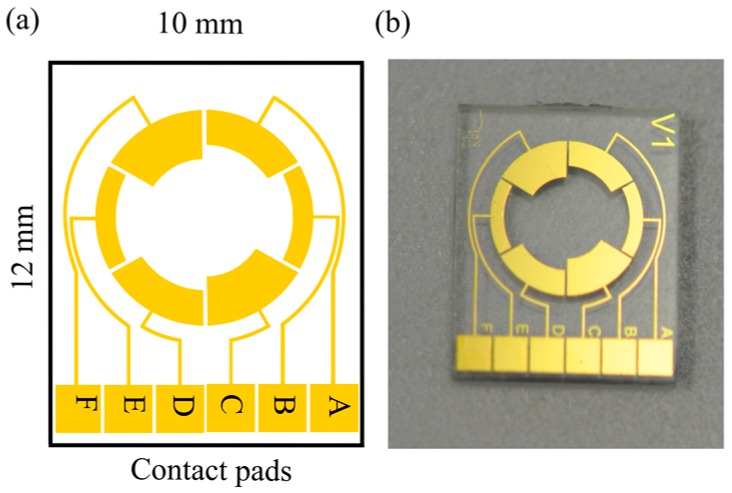
(**a**) Schematic view of the sensor design with various electrode configurations (AD, BE, CF) used for the measurements of impedance changes in the culture medium due to the mold growth; (**b**) optical image.

The detection of mold is carried out by an integrated approach of impedance and colorimetric principles. The sensor system consists of electrodes for measuring the impedance changes in the culture medium as the mold grows and a color sensor for detecting the color transition that takes place at specific (known) pH values. The impedance of the culture medium is measured by using a symmetrical two-electrode configuration implemented on the chip. As the active areas of the electrodes influence the sensitivity of the system, three different electrode configurations (BE, AD and CF) were designed as shown in Figure 2. Three designs have been tested to get more experimental feedback on the circuit-equivalent model and to explain the frequency-dependent behavior of the sensor. The active area of the working electrodes for different configurations are 2.5 mm^2^ (BE), 3.5 mm^2^ (AD) and 4.5 mm^2^ (CF). The electrodes have a distance of 3.5 mm, 4.5 mm, and 5.5 mm between the electrode edges for the CF, AD and BE configurations, respectively. By fabricating all the three electrode configurations on the same chip, their performance could be compared with the same medium, at the same time. For further measurements, only the best performing configuration will be presented. The open area in the middle of the sensor is used for measuring the color of the medium optically. The dimensions of the sensor are 10 mm × 12 mm and a glass cavity with a diameter of 7 mm was glued onto the substrate which acts as a reaction chamber with a volume of 400 µL. 

As it is not possible to assign an absolute value of pH for the measurements performed with the impedance sensor without pre-calibrating the sensor to determine offset values and sensitivity (impedance change per pH-unit), a standard pH indicator dye (that changes color at specific pH values) has been added to the culture medium as a reference. When using methyl red, the color of the medium will be orange at pH values below 5.5 and yellow for pH values above 6.5. In between those values the color transition takes place. The color of the medium is monitored with a color sensor (so-called RGB measurement where the intensities of red, green and blue are determined). Once these pH values have been assigned to the measured impedance, further shifts in the pH can then be determined using the impedance readings. 

The culture medium is prepared with malt extract (X976.1, Carlroth), agar powder (6494.1, Carlroth) and the pH indicator dye (5003.1, Carlroth, methyl red) in deionized water. This mixture is sterilized using an autoclave (15 min, 121 °C at 250 kPa) and a volume of 200 µL is transferred to the reaction cavity while it is in liquid form at 45 °C under sterile conditions in a laminar flow cabinet. Once transferred, this mixture is allowed to solidify at room temperature resulting in a gel. For our experiments, mold spores with different concentrations are transferred into the reaction cavity using a pipette. The mold detection is done by measuring the changes in the impedance of the culture medium due to the changes in the pH as the mold grows. This change in the pH of the medium is caused by the metabolic activity of the mold. As the mold grows, it utilizes the organic matter present in the culture medium and releases some secondary metabolites as by-products that change the pH of the medium [27].

### 2.2. Fabrication of the Mold Sensor

**Figure 3:**
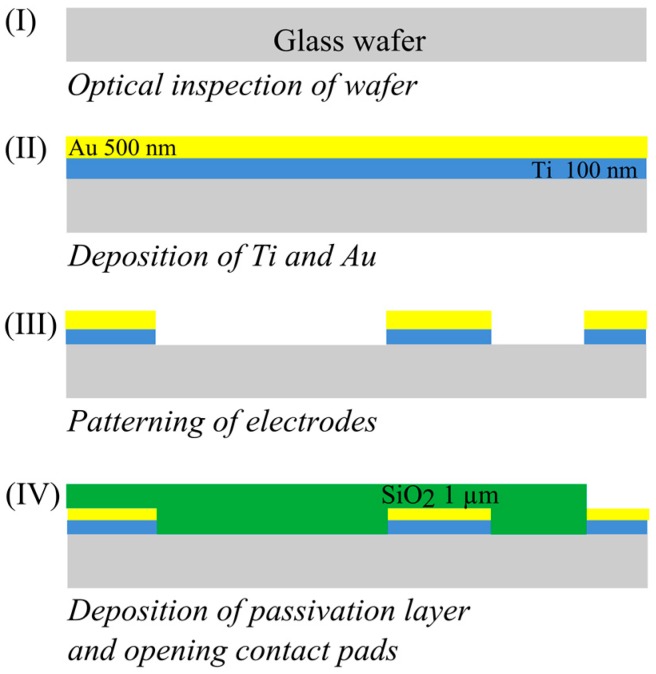
Schematic of the sensor fabrication process.

The fabrication process of the designed sensor on a glass wafer is depicted in Figure 3. The fabrication starts with the deposition of a layer of titanium with a thickness of 100 nm using a physical vapor deposition (PVD) process. On this layer a 500 nm gold layer was sputtered. The titanium layer is used to promote the adhesion of gold to the substrate. Patterning of the electrodes was done using photolithography and wet chemical etching. Once the electrodes were patterned, 1 µm of silicon dioxide was deposited using plasma enhanced chemical vapor deposition (PECVD). This layer acts as a passivation layer, isolating the electrodes. Contact pads were opened by etching SiO_2_ windows.

### 2.3. Reagents, Growth Media and Mold Suspensions

All reagents and chemicals used for the preparation of the mold culture medium were purchased from Carl Roth GmbH, Germany. Mold species that are commonly found in archives and in transport containers like *Fusarium oxysporum*, *Fusarium incarnatum*, *Eurotium amstelodami*, *Aspergillus penicillioides* and *Aspergillus restrictus* were obtained from BMA Labor, Bochum, Germany. Culture mediums with reference pH indicator dyes were prepared under sterile conditions. Mold species were cultured in potato dextrose agar (PDA) and dichloran glycerol agar (DG 18) media at 25 °C for at least 10 days before making suspensions. Spore suspensions were made in sterile deionized water and were diluted to the desired concentrations for using it on chip. An aliquot from the dilution series was diluted further and is plated every time before performing the experiment to determine the actual concentration of the viable spores in the suspension.

### 2.4. Measurement Setup

The impedance between the electrodes was measured using an impedance analyzer (CompactStat, electrochemical type, Ivium Technologies, Eindhoven, The Netherlands). Measurements were done at constant potential in the two-electrode configuration. A sine modulated ac potential of 150 mV was used for the experiments. The impedance was measured at 23 °C in a climatized laboratory (±1 °C, relative humidity 45% ± 5%) in the frequency range of 1 Hz to 1 MHz (Iviumsoft program). A commercial pH meter (Hanna pH 209) was used as a reference for determining the pH of reagents and culture medium. 

**Figure 4:**
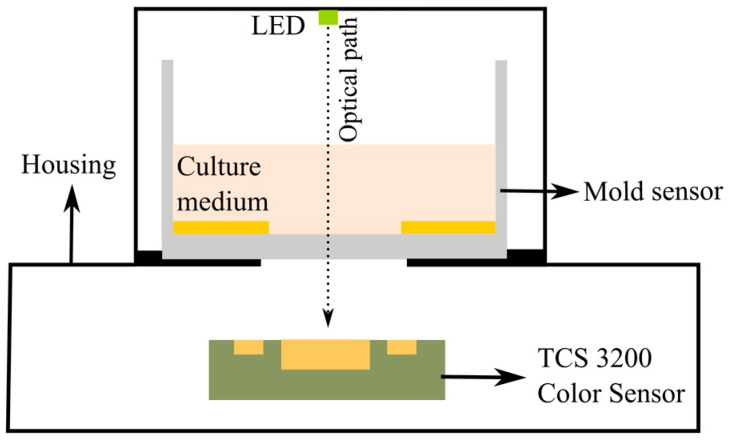
Sensor scheme for detection of color change during mold growth.

**Figure 5:**
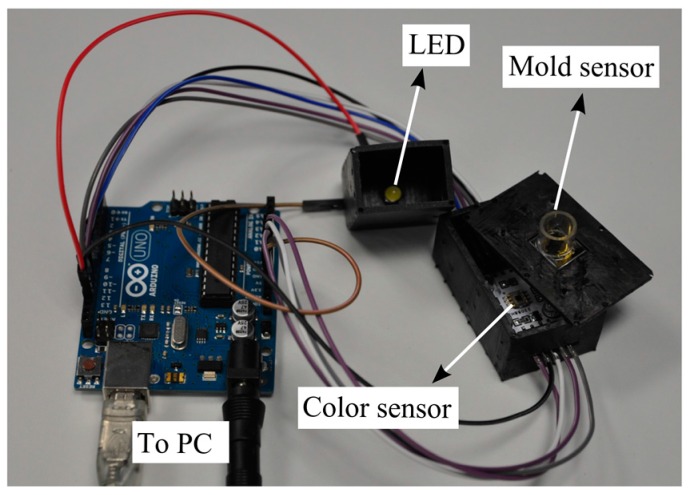
Photo of the experimental setup to measure the color of the medium.

In order to obtain the colorimetric reference measurement, a programmable color sensor TCS3200 (Texas advanced optoelectronic solutions, Plano, TX, USA) was used. This sensor has 64 photodiodes connected in an array format, out of which 16 photodiodes have red filters, 16 photodiodes have green filters, 16 have blue filters and the rest are without filters to measure the intensity of white light. By selecting the red, green or blue filters, the RGB intensity values (in the range from 0 to 255) are determined. The color sensor is placed beneath the mold sensor in a 3D printed outer case. White light is provided from LEDs (model: YSL-A13, Sun LED Technology, Shenzhen, China), which are integrated into the case. For the readouts of the color sensor, a programmable Arduino Uno which is connected to a PC was used. The color sensor is powered (5 V) by the Arduino board. The output of the color sensor is a square wave whose frequency is proportional to the intensity of the light. A schematic view and a photo of the setup are shown in Figure 4 and Figure 5, respectively.

### 2.5. Electrical Equivalent Circuit of the Mold Sensor

**Figure 6:**
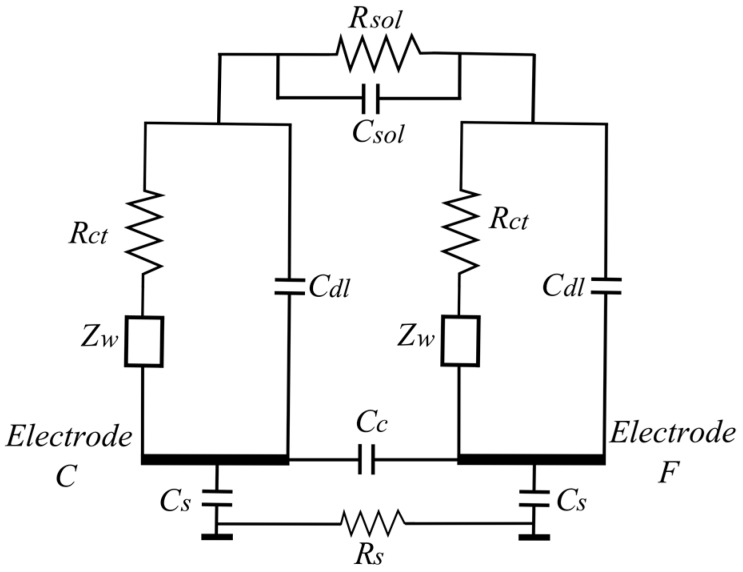
Equivalent circuit model for representation of the impedance measurement applying two electrodes.

It is a common practice to analyze the impedance data obtained from experiments by fitting it to an electrical equivalent circuit model. The model allows the explanation and prediction of the (frequency-dependent) sensor behavior. Several equivalent circuit models are available to represent the physical processes involved during impedance changes. We used the full Randles equivalent circuit, as this model includes the physical processes of charge-transfer and diffusion of charged ions (which happen in the agarose layer during the mold growth). The Randles circuit model for the mold sensor is shown in Figure 6 [28,29,30]. *R_sol_* and *C_sol_* represent the bulk resistance and capacitance of the solution, respectively. *R_ct_* is the charge-transfer resistance at the electrode interface, *C_dl_* is the double layer capacitance that exists at the interface between the electrode and culture medium and *Z_w_* is the Warburg or mass transport impedance. *C_s_* represents the parasitic capacitances between the substrate and the electrodes. *R_s_* is the substrate resistance. *C_c_* is another parasitic capacitance between adjacent electrodes. Additional parasitic capacitances due to the wiring and packaging are not shown in the figure. In brief, the double layer capacitance (*C_dl_*) is formed at the electrode interface due to the separation of charges in the electrode from the ions present in the culture medium. There exists a resistance for the flow of electric charge due to the formation of a double layer at the electrode interface. This interfacial resistance is called the charge-transfer resistance (*R_ct_*) and depends on the double layer composition. For impedance analysis in the frequency range from 1 kHz to 1 MHz, the charge-transfer process is the dominant physical process. Thus, in this frequency the main contribution to the total impedance value is from the charge-transfer circuit parameters (*C_dl_* and *R_ct_*).

The Warburg impedance (*Z_w_*) represents the mass transport impedance due to the diffusion of charged ions towards the electrode interface. *Z_w_* is high at lower frequencies (i.e., <1 kHz) where diffusion processes dominate. In cases where conductive or semiconductor material (e.g., silicon) is used as the substrate, there exists a substrate capacitance (*C_s_*) between the electrodes and the substrate. If the substrate is an insulator such as glass, the substrate capacitance can be neglected. The elements of the circuit model are calculated by fitting with the experimental results. 

## 3. Results and Discussion

### 3.1. Influence of Reference pH Indicator Dye on Mold Growth

**Figure 7:**
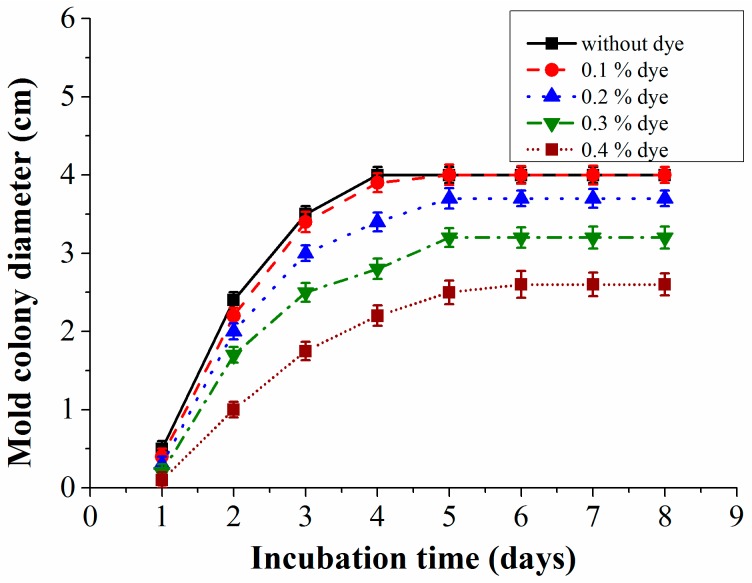
Growth curves of mold species with varied methyl red dye concentrations measured at room temperature (23 ± 1°C).

In our experiments, methyl red dye has been used as a reference pH indicator dye. Not much information is available regarding its toxicity to molds but this dye has been proved to show acute toxicity for aquatic organisms [31]. To ensure that this indicator dye does not inhibit the growth of the mold species, experimental investigations were done to determine the best suitable concentrations of the dye (Figure 7). Also with this test, the highest concentration of methyl red that can be used in the system is determined because at a higher concentration it is easier to measure the color changes. Experiments were started by preparing various concentrations of the indicator dye in the culture medium. All the investigated mold species were grown on petri dishes with varied dye concentrations. The colony diameter was recorded to determine the influence of the indicator dye’s concentration on the mold growth, which is represented graphically in Figure 7. Measurements were repeated three times and the results show that a concentration of 0.1% is just acceptable because it inhibits the growth of the mold only slightly when compared to its growth on a medium without any dye. Higher concentrations seem to be toxic for the mold. 

### 3.2. Characterization of the Mold Sensor

**Figure 8:**
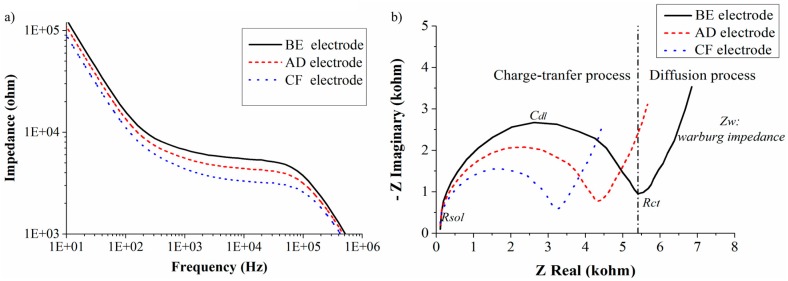
(**a**) Impedance response represented in Bode plot for 200 µL of PDA agar medium measured with different electrode configurations; (**b**) Impedance response in Nyquist plot for 200 µL PDA agar medium measured with three electrode configurations. Vertical dotted line shows the separation of physical process for BE electrode configuration. Both plots show the frequency range of 1 Hz to 1 MHz.

We have tested the three different electrode designs (BE, AD, CF). The active areas of these electrodes are 2.5 mm^2^, 3.5 mm^2^, and 4.5 mm^2^, respectively. The surface area has an influence on the value of *C_dl_* and *R_ct_*. The different distances between the electrode pairs influence *R_sol_* and *C_sol_*. The sensor is characterized by loading 200 µL of PDA agar medium, which has an initial pH of 5.5 (±0.2). The impedance response of all three electrode pairs was measured and represented graphically in Figure 8a (Bode plot) and Figure 8b (Nyquist plot). The Bode plot shows detailed information of the impedance versus frequency. The Nyquist plot is used to get insight into the conduction mechanisms taking place in the culture medium. From Figure 8a one can observe that the measured impedance has a frequency-dependent behavior. This behavior can be explained using the following electrical equivalent circuit model.

There exist three characteristic frequency regions that can be classified based on the circuit element that provides maximum contribution to the total impedance and also the physical process involved. In the low frequency region (i.e., <1 kHz), diffusion processes play a major role. In this region the Warburg impedance is the major contributor to the total impedance. In the medium frequency range (i.e., from 1 kHz to 100 kHz) where the charge-transfer process plays a significant role, the major part of the impedance is contributed by both the double layer capacitance and the charge-transfer resistance. These impedance contributions from *C_dl_* and *R_ct_* are represented as a semicircle in the Nyquist plot. In the high frequency region (i.e., >1 MHz), the solution resistance dominates the impedance. The growth of mold changes the ionic concentration of the culture medium. This change is studied by observing the changes in *C_dl_* and *R_ct_*. The diffusion impedance is observed to be constant as the mold grows in the culture medium. Therefore, the charge-transfer phenomenon in the medium frequency region is the major contributor to the impedance. 

In Table 1 we compare the values of the circuit components determined by feeding the experimental results into the model. The measured data has been fitted using the Iviumsoft program and the obtained fitting parameters for the circuit model are represented in Table 1. The electrode with the largest surface area (CF) has a high double layer capacitance (30 nF), which simultaneously results in a low charge-transfer resistance (3.7 kΩ). The electrodes with the smaller surface area and higher electrode distances (AD and BE) have lower double layer capacitances (24 nF and 19 nF) and higher charge-transfer resistance (4.8 kΩ and 6.0 kΩ), respectively. Because of this the total impedance of the CF electrode pair is lower compared to the other two electrode configurations, AD and BE (see Figure 8a). In our work glass is used as a substrate material. As glass is an insulator, the parasitic substrate capacitances (*C_s_*) and substrate resistances can be neglected. Also, the values of other parasitic capacitance (*C_c_*) and solution capacitance (*C_sol_*) are negligible compared to the double layer capacitance (*C_dl_*). The solution resistance (*R_sol_*) will also change, but because the change is much smaller than the double layer resistance, it does not influence the overall impedance very much.

To determine the sensitivity of each electrode configuration, the pH of the medium has been lowered by 1 unit by adding citric acid. The addition of citric acid increases the number of polar molecules inside the medium, which results in the enhancement of dielectric permittivity, decreasing the double layer thickness. Thus, the addition of citric acid increases the double layer capacitance and lowers the charge-transfer resistance as shown in Table 1. Variation in the pH from 5.5 to 4.5 results in the increase of double layer capacitance of CF electrode from 30 nF to 36 nF, which makes the charge-transfer resistance to decrease from 3.7 kΩ to 3.0 kΩ. This results in a change of 20% in the impedance per unit pH change. If AD and BE electrodes are used then the double layer capacitance rises from 24 nF and 19 nF to 28 nF and 21 nF, respectively. Simultaneously, the value of *R_ct_* decreases from 4.8 kΩ and 6.0 kΩ to 4.1 kΩ and 5.5 kΩ, respectively. Thus, for the AD and BE electrode configurations there is a 15% and 10% change in impedance per unit pH change. Of all the three electrode configurations designed in our work, the CF electrode design shows the highest sensitivity with a 20% impedance change.

### 3.3. Detection of Mold Growth Using Impedance and Colorimetric Measurements

**Figure 9:**
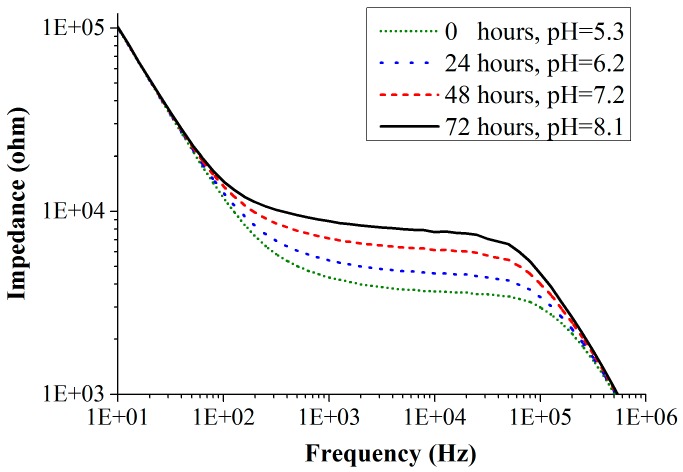
Impedance spectra of the mold sensor recorded during the growth of *Eurotium amstelodami* mold species measured with CF-electrodes.

The impedance spectra of one of the mold species *Eurotium amstelodami* measured on chip in the frequency range of 10 Hz to 1 MHz for an initial inoculum concentration of 10^3^ CFU/mL (colony forming units/mL) is shown in Figure 9. Experiments were performed with all the three electrode configurations. For a clear understanding, we have shown the impedance response curves using the CF electrode configuration only. Figure 9 shows the change in the impedance of the medium as the mold grows. With the growth of mold, nutrients in the culture medium are metabolized, changing the pH (i.e., increase) of the medium. As the pH increases, there is a decrease in the amount of polar molecules present in the culture medium. This results in the decrease of the double layer capacitance, which simultaneously increases the charge-transfer resistance. Correspondingly, the impedance of the culture medium increases with time as shown in Figure 9. This graphical behavior of the sensor has been explained with the Randles electrical equivalent circuit in the previous Section 2.5. From the fitted data, one can observe that the initial values of the double layer capacitance and charge-transfer resistance before the mold growth are 30 nF and 3.7 kΩ, respectively. After 24 h of mold growth, the double layer capacitance decreased to 24 nF and the charge-transfer resistance is increased to 4.4 kΩ. Growth of the Eurotium species shows proportional impedance change (absolute value of impedance) of 23% in 24 h (measured at 10 kHz), and the measurements were done for 72 h. A total of six measurements were performed for each species and all of them show similar results with a standard deviation of lower than 10%. Similar behavior in the impedance curves was observed for the measurements performed with AD and BE electrode configurations. They both show somewhat lower impedance changes, as expected (because of low sensitivities).

The ionic concentration of the culture medium in the reaction chamber changes the values of *C_dl_* and *R_ct_*. Thus, the measured impedance values will also change. If the volume of the culture medium changes, *C_dl_* and *R_ct_* are not affected but *R_sol_* will be. However, because *R_sol_* is much smaller than the double layer impedance, the influence of it on the measurement is not that large. The measured impedance values depend on the ionic concentration of the culture medium in the reaction chamber. From Figure 6 it can be seen that if there is a change in ionic concentration, the values of *C_dl_* and *R_ct_* will change. 

**Figure 10:**
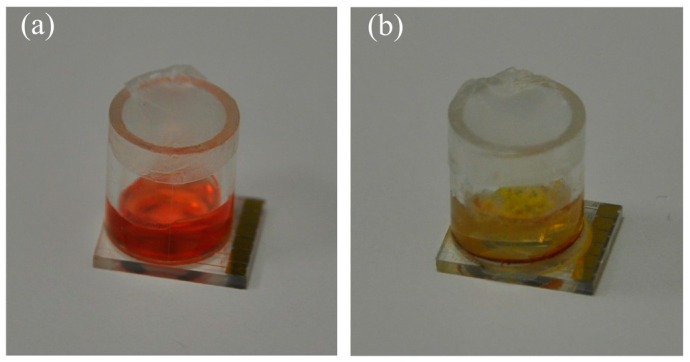
Culture medium prepared using methyl red as a reference indicator dye: (**a**) initial orange color of the medium in the cavity where pH is 5.5; (**b**) medium turned yellow due to increase in pH to 6.5 with the growth of *Eurotium* mold. The glass cavity has an internal diameter of 7 mm.

To include a reference value for pH and also to determine the sensitivity of the impedance to the pH change, an independent colorimetric reference measurement is added. A pH indicator dye added to the culture medium changes the color of the medium within a defined pH range. Figure 10 shows this principle where the culture medium has been prepared with a methyl red indicator dye. The medium with this indicator has an initial color of orange at pH 5.5 when no mold is growing and changes its color to yellow once the pH of the medium has been increased to 6.5 with the growth of mold. Thus, by measuring the color change of the medium with a color sensor, it is possible to determine the absolute change in the pH.

**Figure 11:**
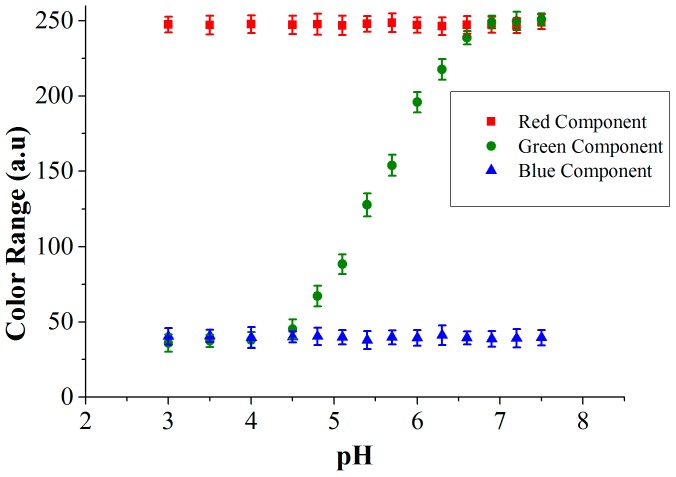
Measured RGB values from the color sensor for the methyl red indicator dye at different pH.

The RGB values of the culture medium with methyl red indicator dye for different pH values measured using the color sensor are shown in Figure 11. The variation in the RGB intensities depends on the dye used in the culture medium. With the methyl red indicator dye, the color of the medium changes from red to yellow (in the pH range: 4.5–7.0) and this transition in the visible spectrum is due to the change in green component. We depict all three RGB components in Figure 11 to show that red and blue are not changing, which is also color information. To determine the sensitivity of the sensor, a unit change in pH was measured using the TCS 3200 color sensor (Texas advanced optoelectronic solutions, Plano, TX, USA) and this value is assigned to the corresponding impedance change. Once the sensor is calibrated using the reference colorimetric method, further shifts in the pH are determined by impedance changes. A relation of 0.9 kΩ/pH unit was determined for the part in the pH curve (Figure 11), where both optical and impedance methods are active (roughly between pH 5.5 to 7.0). As the pH of the medium crosses 7.0, the optical data saturates.

In the described mold growth experiment, we measured a green value of 118 that changes to a value of 210 after 24 h. Taking now the curve in Figure 11, we find the interpolation that the pH for the value 118 corresponds to 5.3, and for the value 210 it matches a pH of 6.2. The corresponding impedance measurements were 3.65 kΩ (at pH = 5.3) and 4.5 kΩ (at pH = 6.2). This results in an impedance change of 23%. The sensor performance is determined by implementing different mold species that are common contaminants found in archives and in food transport containers. Three different species, *Fusarium oxysporum*, *Fusarium incarnatum* and *Eurotium amstelodami*, grow relatively quickly and produce pH changes from 5.3 to 8.5 with a sensitivity of 0.9 kΩ/pH unit (for initial concentration of 10^3^ CFU/mL and measured with CF-electrode configuration), whereas other species that are normally found in archives (*Aspergillus penicillioides* and *Aspergillus restrictus*) produce a smaller pH change from 5.3 to 7. The impedance change is used for the quantification of the initial spore concentration of the mold in the culture medium using the integrated approach. 

**Figure 12:**
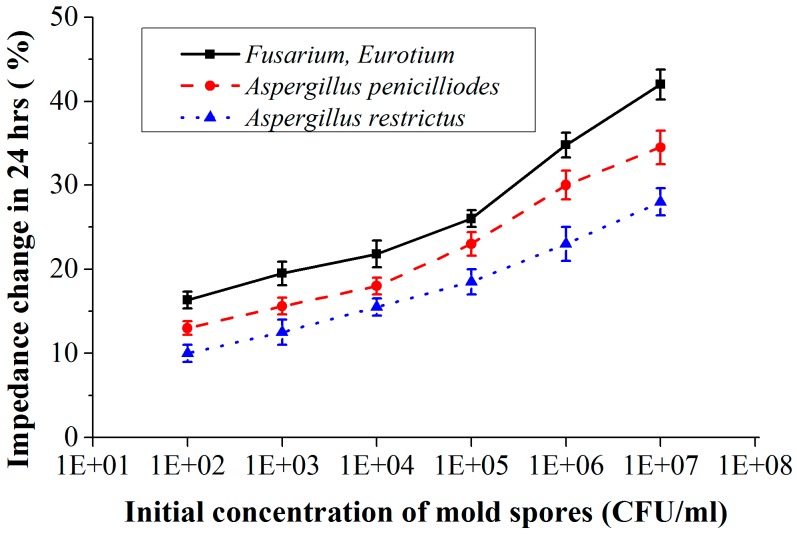
Percentage of impedance change as a function of initial concentration of the mold spores for a detection time of 24 h (impedance measured with CF-electrode at 10 kHz).

Figure 12 shows the measured results where the percentage of impedance change is plotted as a function of the initial concentration of mold spores for a constant detection time of 24 h (impedance measured at 10 kHz, error bars indicate the standard deviation calculated from 6 measurements). At higher initial spore concentrations the change in impedance occurs more quickly, resulting in higher change in impedance for a constant detection time. For example, if the initial spore concentration of *Aspergillus restrictus* is 10^3^ CFU/mL then in 24 h the impedance of the culture medium will be changed by 12% (measured at 10 kHz), whereas if the concentration is 10^6^ CFU/mL, then the impedance changes by 23% (measured at 10 kHz) in 24 h. Also, from the results it is clear that different mold species with same initial concentration could produce different impedance changes. Although we do not aim at differentiating between the molds, the quantification results (Figure 12) show that different mold species with the same initial concentration have different impedance change rates. Thus, if growth rate value differences are large enough, this could be used to give a first indication of the mold type. Another method could be to apply mold selective culture media. This is not a part of this study, however.

In the applications mentioned, archives and food containers, a time response of 24 h or better is desired for the detection of mold. The quantification results (Figure 12) show that the achieved sensitivity with the CF electrode configuration is enough to determine the initial concentration of spores within 24 h. With the results indicating that the sensor could detect a concentration of 10^2^ CFU/mL, the designed sensor is sensitive enough for usage in fruit logistics and in archive environments where typical contamination concentration of 10^3^ CFU/mL–10^4^ CFU/mL is treated as a risk for its environment [32]. Another advantage of the method is that it can easily be automated by integrating an air sampler.

## 4. Conclusions and Outlook

A miniaturized culture medium-based sensor using an integrated approach with impedance and colorimetric techniques for the detection of mold growth has been successfully demonstrated. The colorimetric principle serves as the reference measurement in the system, with which the absolute value of pH and the sensitivity of the sensor per unit change in pH are determined directly on-chip without the need to pre-calibrate the sensor. Tests were performed with major mold species responsible for contaminations in the archives and in the food transport containers, i.e., *Fusarium oxysporum*, *Fusarium incarnatum*, *Eurotium amstelodami*, *Aspergillus penicillioides* and *Aspergillus restrictus*. 

This sensor offers the advantages of low cost and ease of automation for logistic, archive and indoor applications. By integrating an air-sampling unit and read-out electronics, the proposed sensor is highly promising for making a fully automated mold monitoring system to screen mold contaminations in fruit containers and archive environments.

## Figures and Tables

**Table 1:** Fitting parameters for the circuit model of three electrode configurations at different pH values.

Parameter	CF-Electrode Design		AD-Electrode Design		BE-Electrode Design	
pH	5.5	4.5	5.5	4.5	5.5	4.5
*R_ct_*: kΩ	3.7	3	4.8	4.1	6	5.5
*C_dl_*: nF	30	36	24	28	19	21
